# Visualization and bibliometric analysis of cAMP signaling system research trends and hotspots in cancer

**DOI:** 10.7150/jca.47158

**Published:** 2021-01-01

**Authors:** Caoli Tang, Duanya Liu, Yongsheng Fan, Jun Yu, Cong Li, Jianmei Su, Chunhong Wang

**Affiliations:** 1 Department of Preventive Medicine, School of Public Health, Wuhan University, Donghu Road 115, Wuhan 430071, Hubei, China.; 2 Key Laboratory of Regional Development and Environmental Response, Faculty of Resources and Environmental Science, Hubei University, Friendship Avenue 368, Wuhan 430062, Hubei, China.

**Keywords:** cAMP, Cancer, CiteSpace, Bibliometrics, Hotspots

## Abstract

Cyclic adenosine monophosphate (cAMP) is an essential second messenger that widely distributed among prokaryotic and eukaryotic organisms. cAMP can regulate various biological processes, including cell proliferation, differentiation, apoptosis and immune functions. Any dysregulation or alteration of cAMP signaling may cause cell metabolic disorder, immune dysfunction and lead to disease or cancer. This study aimed to conduct a scientometric analysis of cAMP signaling system in cancer field, and explored the research trend, hotspots and frontiers from the past decade. Relevant literatures published from 2009 to 2019 were collected in the Web of Science Core Collection database. EndNote X9 was used to remove duplicate articles, and irrelevant articles were manually filtered. Bibliometric analyses were completed by CiteSpace V. A total of 4306 articles were included in this study. The number of related literatures published each year is gradually increasing. Most of them belong to “Biochemistry & Molecular Biology”, “Oncology”, “Cell Biology”, “Pharmacology & Pharmacy” and “Endocrinology & Metabolism” areas. In the past decade, USA, China, and Japan contributed the most to the research of cAMP signaling system in cancer. The frontiers and hotspots of cAMP signaling pathway system related to cancer fields mainly focused on cancer cell apoptosis, metastasis, and multiple tumors occurrence in patients with Carney complex. Intervention of the cAMP metabolic pathway may be a potential and promising therapeutic strategy for controlling clinical cancer and tumor diseases.

## Introduction

Cyclic adenosine monophosphate (cAMP) is an important cellular second messenger that firstly discovered in 1956 1 and widely distributed among prokaryotic and eukaryotic organisms. cAMP mediates a multitude of cellular processes and biological functions 2, including gene regulation, cell proliferation and differentiation, apoptosis, energy metabolism, memory formation, neurotransmission, intestinal secretion, retinal phototransduction and immune functions 3-9. cAMP is formed from ATP by adenylyl cyclases (ACs) and hydrolyzed by phosphodiesterases (PDEs) 10,11. cAMP exerts its actions by targeting the downstream effectors such as cAMP-dependent protein kinase (PKA), exchange proteins directly activated by cAMP (EPACs), and cyclic nucleotide-gated ion channels (CNGC). PKA is a major effector that can subsequently phosphorylate cAMP response element binding protein (CREB). CREB is an essential transcriptional co-factor which initiates an array of transcriptional cascades and target gene expressions 12.

Given the crucial roles of cAMP plays, perturbing the cAMP signaling processes may cause cell metabolic disorder and multiple organism diseases. Cancer is one of the tumor development diseases caused by abnormal cell proliferation and differentiation, mainly presents as apoptosis inhibition and insufficient cell death. Current progress in cancer field has disclosed a lot of rational methods in dealing with tumor formation 13-16, and many scientists have turned their attention to the universal small organic molecule cAMP, which influences vital cellular signaling transduction and regulates physiological metabolic processes 17-21. Therefore, cAMP has become an ideal target for contemporary therapies for cancer and tumor diseases, as well as combating bacteria virulence and developing new drugs. Although numerous approaches seek to target the cAMP signaling system, such as synthanses, hydrolyases, and downstream effector proteins and RNAs, the precise molecular mechanism of cAMP causes or defenses against cancer is still relatively unclear and the clinical treatment effect is also less reported. Bibliometrics has been widely used in the analysis of research output, as well as the discovery of hotspots and research trends 22. So bibliometrics is helpful to understand the knowledge base and research frontier of a specific field. In addition, CiteSpace which is developed by Chaomei Chen is an effective visualization software based on bibliometrics 23,24. However, there is no bibliometric study on cAMP signaling system in the cancer field to date. In this study, the research literatures on the role of cAMP signaling system in the field of cancer published in the past decade were retrieved from the Web of Science and analyzed by the bibliometric study. The purpose of this scientometric study is to analyze the research literatures of cAMP signaling system with its linkage to cancer, and explore the research trends, hotspots and other useful information in this field.

## Materials and Methods

### Data source and search strategy

Relevant literatures were collected in the Web of Science Core Collection (WoSCC) database which was widely used in bibliometric analysis 25,26. The search query was “TS (Topics)= (“cAMP” or “cyclic adenosine monophosphate” or “cyclic AMP” or “protein kinase A” or “PKA” or “EPAC*” or “CNGC”) and TS= (“tumor*” or “cancer*” or “carcino*” or “onco*”)” (the symbol “*” is a retrieval symbol of WOS advanced retrieval, which means to search for words prefixed with the word before “*”). Research results were restricted by language (English) and document type (Article). Publication year was restricted from 2009 to 2019. Literature search was completed within a day to avoid bias caused by database updates. A total of 4590 articles were retrieved. Considering that the word “camp” has the meaning of vacation camp, the retrieved literature was screened, and the articles that the definition of “camp” is not cyclic adenosine monophosphate were excluded. 284 articles and duplicate literature were screened out, so a total of 4306 articles were included in this analysis.

### Data analysis tools

EndNote X9 was used to remove duplicate articles, and then irrelevant articles were manually filtered. CiteSpace V was used to complete bibliometric analyses of categories, countries and institutions, co-cited journals, authors and co-cited authors, co-occurrence of keywords and references. The parameters of CiteSpace were set as follows: time slicing (from 2009 to 2019, one year per slice); term source (title, abstract, author keywords, keywords plus); node types (select author, institution, country, keyword, category, reference, cited author and cited journal in turn); strength (cosine); scope (within slices); selection criteria (top 50).

The visualization map is mainly composed of many nodes and interwoven connections. Nodes represent different subject categories. The size of each node indicates the number or citation frequency. The larger the circle, the more literature belongs to this research field. Each circle is made up of different color rings, and each color corresponds to the time scale. The change of color from cold blue tone to warm red tone in the time scale indicates the change of time from early to recent. If the periphery of a node is bright red or bright purple, it represents that the node is a burst node or its betweenness centrality is bigger than 0.10. Betweenness centrality measures the degree of one node in the middle of a path that connects to other nodes in the network. The nodes with high centrality are located in the center of the network, which are the transitions of other parts. The line which links two circles indicates that they are connected or cooperated or co-cited. The thickness of a line is proportional to its strength or correlation 27,28.

## Results and Discussion

### Analysis of the basic situation of the extracted literature

#### Analysis of publication outputs

**Figure 1:**
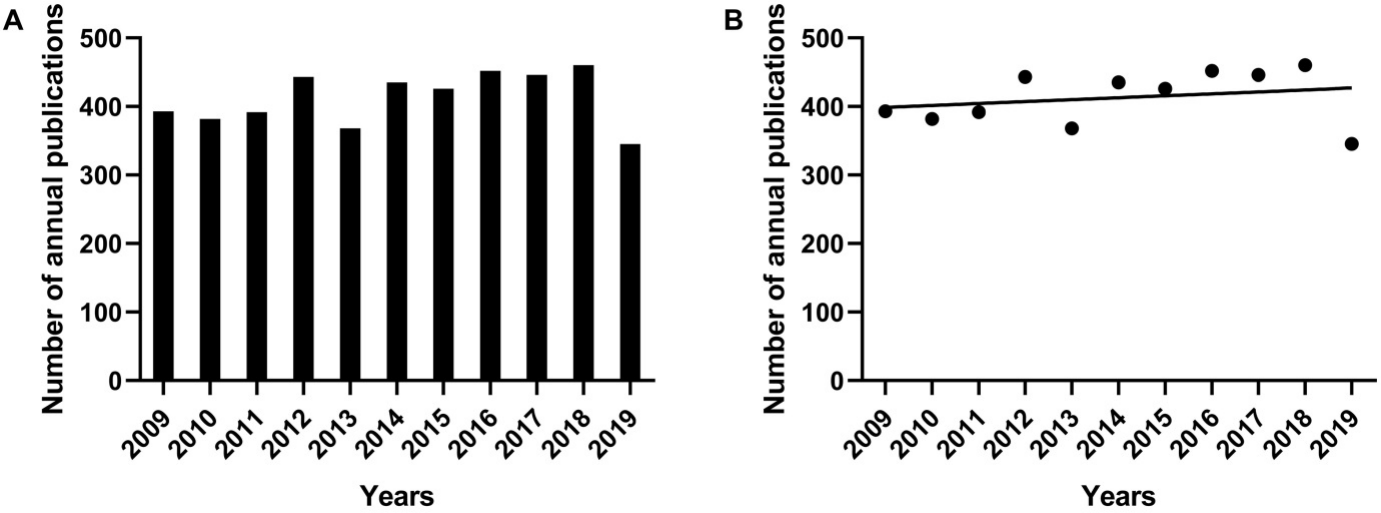
The number of annual publications on the research of cAMP signaling system with its linkage to cancer from 2009 to 2019. (A) Histogram of the number of annual publications. (B) Scatter plot and trend line of the number of annual publications.

In this study, 4306 articles were included. The number of annual publications was generally considerable, but the number in the year of 2013 and 2019 was relatively small compared with that of other years (**Figure 1A**). There was no obvious research trend but kept relatively stable from 2009 to 2019. In order to understand the general research trend of cAMP signaling system with its linkage to cancer in the past decade, the trend line of publications in the scatter plot was drawn in **Figure 1B.** The trend line was stable with a slight upward tendency. Based on these results, it can be inferred that the corresponding research is in a plateau period in recent years. As is known to all, the topic of cancer has always been a hotspot in various fields for a long time, while the related research of cAMP signaling system is also at a mature stage, there may be some new breakthroughs in the next few years.

#### Analysis of categories

**Figure 2:**
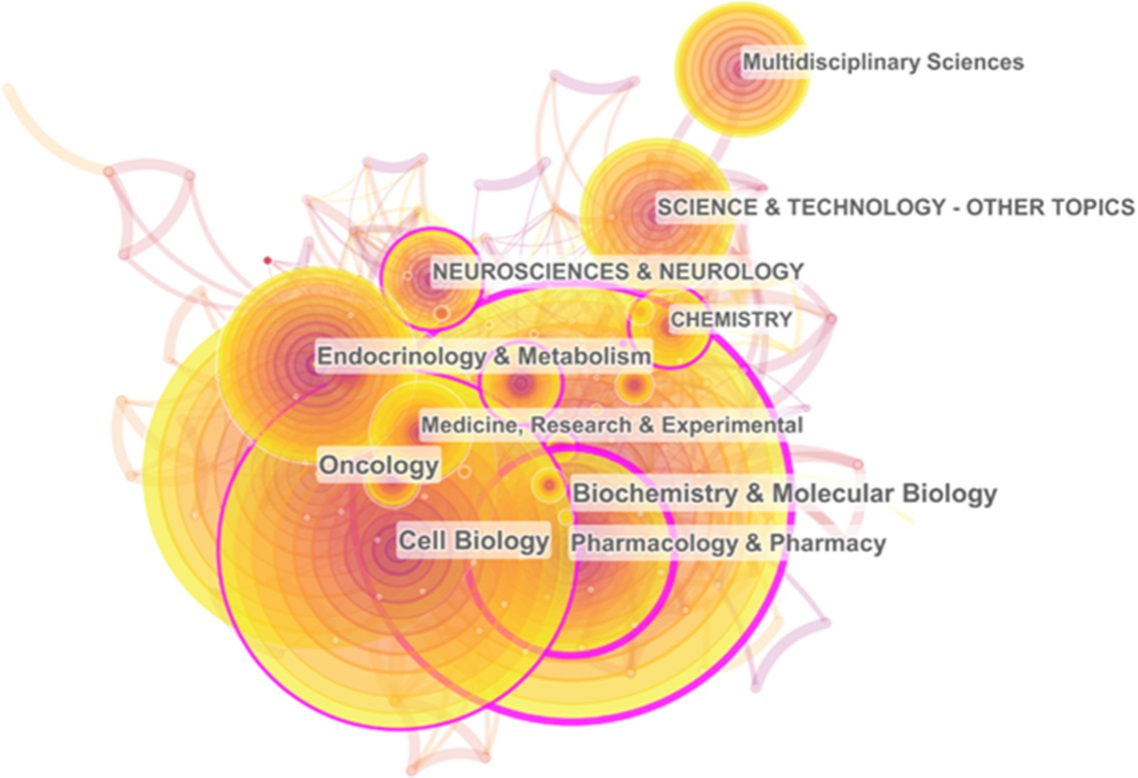
The co-occurrence network of subject categories.

CiteSpace's co-occurrence analysis of subject categories, which is based on Web of Science category, is an effective way to understand the related research fields. Visual calculation results show that these 4306 papers mainly appear in 105 research fields. **Table 1** shows the ranking of subject categories according to the publication frequency and betweenness centrality, respectively. “Biochemistry & molecular biology” (997; 0.25) accounted for the largest proportion and had the second highest centrality. In addition, “Pharmacology & Pharmacy” (481; 0.27), “Cell Biology” (823; 0.11), “Neurosciences & Neurology” (254; 0.14) and “Chemistry” (214; 0.15) also had a high frequency and centrality. **Figure 2** shows the co-occurrence network of subject categories. The nodes of “Biochemistry & Molecular Biology”, “Pharmacology & Pharmacy” and “Cell Biology” are relatively large, and the outer rings of these circles are purple, indicating that most of the researches of cAMP signaling system in the cancer field focus on these three directions. Thus, studies in these research areas are of great significance to other areas.

#### Analysis of leading countries and institutions

**Figure 3:**
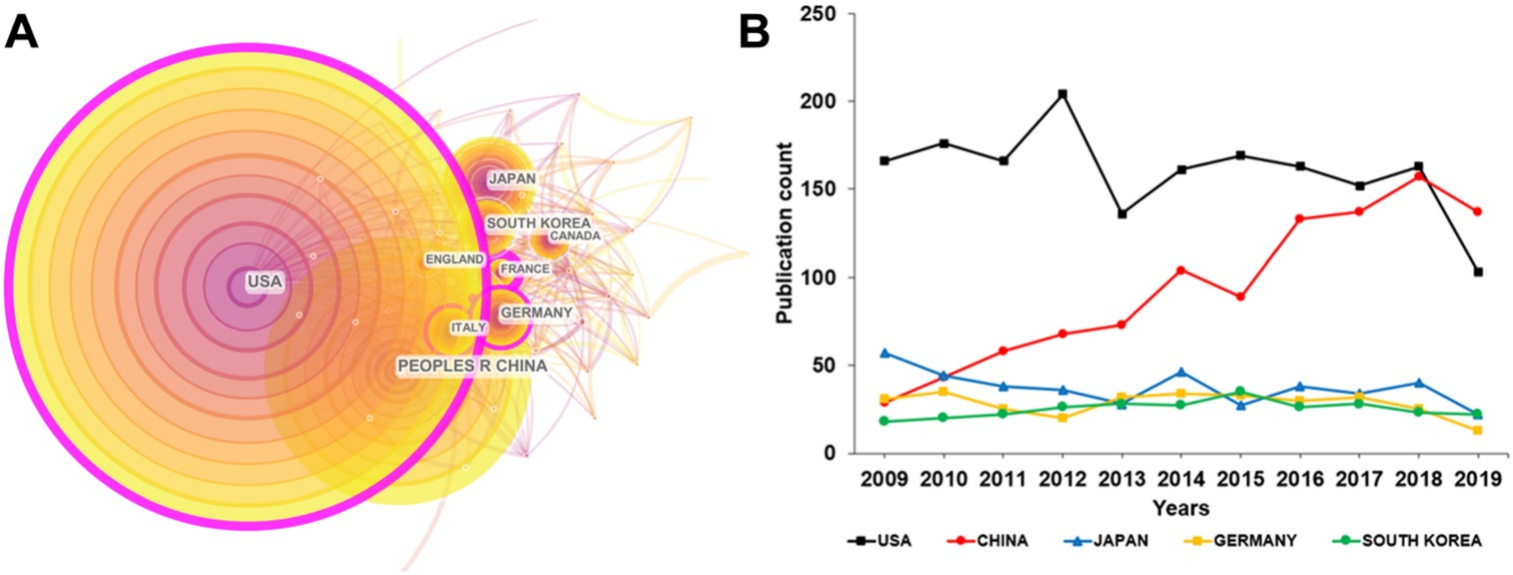
(A) Collaboration network map of leading countries for cAMP signaling system with its linkage to cancer from 2009 to 2019. (B) Time-varying trend of the number of published publications in the top five countries each year.

These publications were contributed by 86 countries. **Figure 3A** with 57 nodes and 389 links shows these authors' countries and their collaboration network. The circles and lines in **Figure 3A** respectively represent each country's output and their collaboration. United States of America (USA) was the most productive country with 1758 publications. The People's Republic of China (China) ranked second with 1028 publications. Japan (410), Germany (310) and South Korea (275) ranked third to fifth. To sum up, USA, China, Japan, Germany, South Korea, Italy, Canada, France and England played a major role in the research of cAMP signaling system with its linkage to cancer.

Each country had a different publication trend. **Figure 3B** displays the time-varying trend of the annual publications in the top five countries. Although the largest amount of the publications was contributed by USA, it performed a decreasing trend after 2012, while China showed a continuous upward trend over time. It can be predicted that in the next few years, China will surpass USA in the number of publications and leap to the top. The reasons why a developing country can increasingly publish a huge quantity of research on cAMP with its linkage to cancer are as follows. Firstly, Chinese government's investment in scientific research, especially the basic medical research is increasing. Secondly, as a country with a large population, China has a substantial absolute number of people suffering from cancer and other diseases, which needs more urgent attention and treatment.

There are 3654 institutions involved in these publications. As shown in **Table 2**, the most productive institution was Chinese Academy of Sciences (65), followed by Shanghai Jiao Tong University (60), University of Texas MD Anderson Cancer Center (56), Seoul National University (54) and University of California- San Diego (49) respectively. Among the top ten institutions according to the number of publications, most were from China and USA, which corresponded to the distribution of the country. Figure S1 shows the collaboration network among these institutions. From the perspective of inter-agency cooperation, there was closer cooperation between institutions within the same country and less collaboration between different countries. The collaboration between USA and other countries was relatively more. National Cancer Institute (0.15), University of Texas MD Anderson Cancer Centre (0.13), University of Pennsylvania (0.13), Harvard University (0.12), Chinese Academy of Sciences (0.11) and University of California-Los Angeles (0.11) were the main centers of institutional collaboration worldwide. If there is more cooperation among institutions from different countries, it may be helpful to make new breakthroughs in cAMP signaling system with its linkage to cancer.

#### Analysis of cited journals

Since the influence of a journal mainly depends on its citation times, the citation analysis of journals reflects the distribution of knowledge base and important research findings in a specific field 29. A total of 76 journals were extracted. **Table 3** presents the top ten cited journals based on the citation frequency, including *Journal of Biological Chemistry* (*J Biol Chem*, 3112), *Proceedings of the National Academy of Sciences of the United States of America* (*P Natl Acad Sci USA*, 2680), *Nature* (2086), *Cancer Research* (*Cancer Res*, 2047), *Science* (1785), *Cell* (1688), *Oncogene* (1511), *Plos One* (1416), *Molecular and Cellular Biology* (*Mol Cell Biol*, 1413) and* Biochemical and Biophysical Research Communications* (*Biochem Bioph Res Co*, 1307). From the perspective of citation frequency and centrality, *J Biol Chem* (3112; 0.42), *Cancer Res* (2047; 0.19) and *P Natl Acad Sci USA* (2680; 0.12) were at the core of these 76 journals. Among the top ten cited journals, *Nature* (43.070) had the largest impact factor (IF), followed by *Science* (41.037) and *Cell* (36.216) respectively. According to the Journal Citation Reports (JCR) 2019 standard, six journals were classified as Q1. Eight of these journals are published in USA and two in England. USA provides a reliable platform for the publication of relevant research papers. Of the 76 journals, 34.21% of their IF (2019) were more than ten, while 22.37% were between five and ten, and the remaining 43.42% were less than five. This result indicates that a relatively large proportion of cited articles are high-quality researches and published in high-IF journals, implying that cAMP signaling is a very hot, interesting and promising field in cancer topic, which has attracted a lot of attentions of scientists all over the world.

#### Analysis of author information

**Figure 4:**
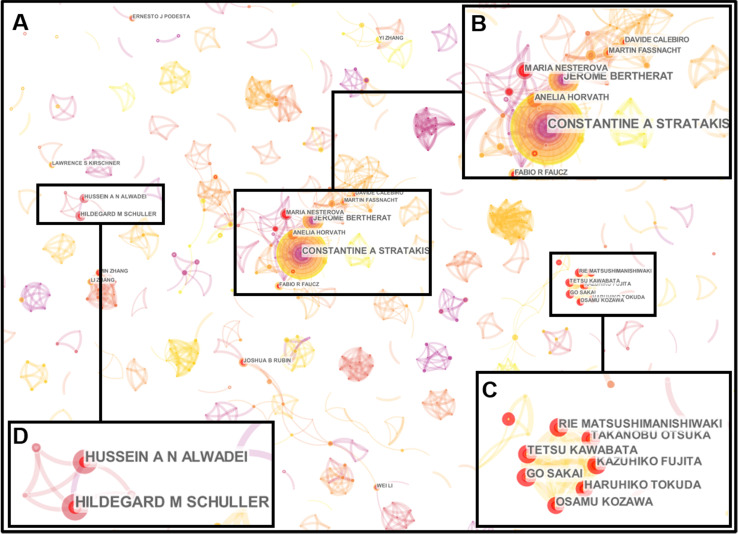
Co-authorship network map. (A) The author collaboration network constructed by CiteSpace was observed with 1036 nodes and 2041 links. Each node represents an author, and each link reflects the collaboration relationship. (B) Stratakis CA, Bertherat J, Nesterova M and Horvath A had a close collaboration from 2009 to 2012. (C) Kawabata T, Fujita K, Sakai G, Matsushima-Nishiwaki R, Otsuka T, Kozawa O and Tokuda H had work together in 2018. (D) Schuller HM and Al-Wadei HA had a connection from 2012 to 2013.

The information of authors and co-cited authors was also analyzed by CiteSpace V. These 4306 articles were produced by 26677 authors. The top ten productive authors and the top ten authors with the largest number of citations were listed in the **Table 4**. Stratakis CA was the most productive author who published 46 articles, followed by Bertherat J (21), Nesterova M (11), Horvath A (11), Zhang W (10), Schuller HM (10), Al-Wadei HA (9), Stocco DM (9), Rubin JB (9) and Kawabata T (8) respectively. Most of them come from USA. However, these top ten high-yield authors together accounted for merely 3.34% of the total literatures, and only six authors had published ten or more articles during the past decade, which indicated that few high-yield researchers had devoted all their energy to the study of cAMP signaling system in cancer field. In addition, some researchers focused on cAMP and cancer studies are due to their cooperation teams, which could be found through the co-authorship network. Although the co-authorship network of this study was very fragmented (**Figure 4A**), some researchers still established collaborations with each other. Stratakis CA, Bertherat J, Nesterova M and Horvath A had a close cooperation from 2009 to 2012 (**Figure 4B**). Kawabata T, Fujita K, Sakai G, Matsushima-Nishiwaki R, Otsuka T, Kozawa O and Tokuda H worked together in 2018 (**Figure 4C**). Schuller HM and Al-Wadei HA had a connection from 2012 to 2013 (**Figure 4D**). In **Figure 4C**, all the authors come from Nagoya City University Graduate School of Medical Sciences and Gifu University Graduate School of Medicine in Japan. Thus, geographical location may be the main reason for the dispersion of cooperative networks.

However, the analysis of co-author relationship only provides the information about authors' outputs and collaboration situation instead of the authors' impact on the cAMP signaling system with its linkage to cancer, which can be reflected by co-cited author analysis. Two or more authors that are cited together are called co-cited authors. As shown in **Table 4**, Livak KJ (166) had the largest number of citation, followed by Mayr B (149), Kirschner LS (114), Wang Y (110), Shaywitz AJ (99), Li Y (94), Jemal A (91), Zhang Y (85), Stocco DM (82) and Taylor SS (75) respectively. Of these top ten co-cited authors, nine come from USA and one from China. This result shows that the research findings of USA scholars provide most abundant knowledge for the research of cAMP signaling system in cancer field. CiteSpace was also used to draw the author co-citation network map (Figure S2). The centrality of five cited-authors including Taylor SS (0.15), Mayr B (0.14), Livak KJ (0.12) Hanahan D (0.12) and Houslay MD (0.11) was greater than 0.1, indicating that they played an important connecting role in the network of co-cited authors. The articles written by Mayr B, Livak KJ and Taylor SS are not only highly cited, but also an important connecting point. For example, a review *“Transcriptional Regulation by the Phosphorylation-Dependent Factor CREB”* 30 written by Mayr B et al. that published in *Nature Reviews Molecular Cell Biology* was cited 1,724 times. This review mainly focuses on some summarized mechanisms that have mediated by CREB signal specifically. Livak KJ et al. 31 introduced the 2^-ΔΔC^_T_ method which may be a convenient and useful method to analyze quantitative real-time polymerase chain reaction data in the article entitled "*Analysis of Relative Gene Expression Data Using Real-Time Quantitative PCR and the 2^-ΔΔC^_T_ Method* ". This method was used in many researches in this field to calculate relative gene expression 32, 33, 34. Taylor SS had an in-depth research on PKA and also published numerous high-quality papers in high impact journals 35, 36, 37.

### Analysis of the basic contents of extracted documents

The frontiers and hotspots of cAMP research in the cancer field can be found by analyzing the results of co-occurrence keywords, co-cited references, burst terms and burst references.

#### Analyses of keywords and burst terms

Keywords are highly refined research content and important indicator to reflect the research theme and hotspots. There were 98 keywords extracted from 4306 articles with 98 nodes and 747 links by CiteSpace (Figure S3). The top 20 most frequently occurring keywords can be divided into four categories, 1) molecules regulating cAMP signaling pathway, including 'cAMP' (761), 'phosphorylation' (420), 'protein kinase a' (354), 'pathway' (320), 'protein' (294), 'receptor' (274), 'gene'(242) and 'CREB' (222); 2) the intervention of cAMP pathway, including 'activation' (650) and 'inhibition' (252); 3) experimental subjects, including 'cancer' (514), 'cell' (368), '*in vitro*' (299), 'breast cancer' (290), 'cancer cell' (233); 4) experimental observation results, including the highest frequency word 'expression' (977), 'gene expression' (482), 'apoptosis' (400), 'proliferation' (334) and 'growth' (328) (**Table 5**). Among them, 'expression' (0.12), 'gene expression' (0.14), 'apoptosis' (0.13) and 'protein kinase a' (0.12) are key nodes, because their betweenness centrality are greater than 0.1, which means they play an important role in linking other keywords.

**Figure 5:**
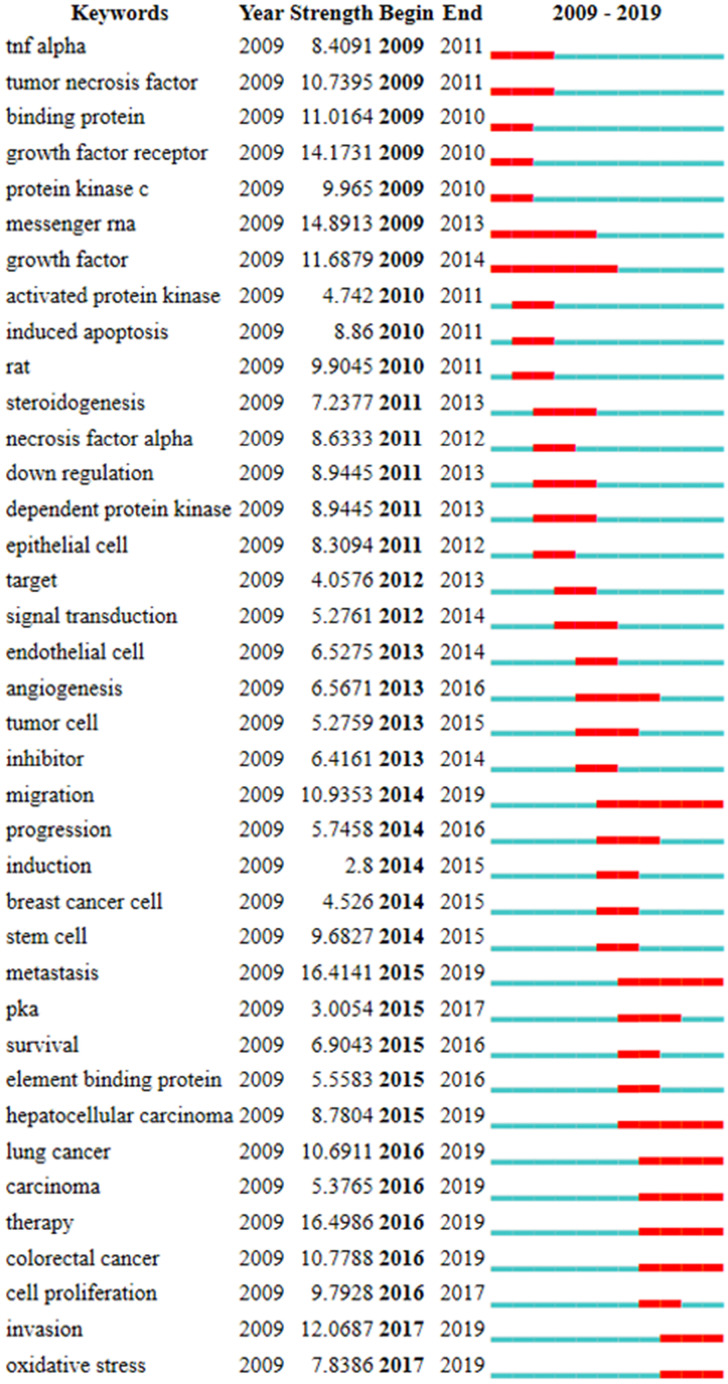
The list of the top 38 keywords with the strongest citation bursts according to their occurrence time. (Note: The red bars mean these keywords cited frequently, while the blue bars mean infrequent citation.)

Burst term refers to a word or an article that appears to rise suddenly over a period of time, which is an important index to deduce the research frontier in the field of characteristic research 38. 38 keywords with the strongest citation burst were extracted in this study. Burst strength and duration are two indexes to evaluate burst terms. **Figure 5** was a sorted list of burst terms according to their occurrence time. Keyword emergence duration was indicated by red bars. These burst terms can be divided into 5 classes: 1) cAMP signaling pathway including 'binding protein', 'protein kinase c', 'messenger RNA', 'dependent protein kinase', 'signal transduction', 'PKA', 'element binding protein'; 2) other molecules that may be affected by cAMP pathway including 'TNF alpha', 'tumor necrosis factor', 'growth factor receptor', 'growth factor', 'necrosis factor alpha'; 3) experimental object including 'rat', 'epithelial cell', 'target', 'endothelial cell', 'tumor cell', 'breast cancer cell', 'stem cell'; 4) experimental treatment including 'activated protein kinase' , 'down regulation' , 'inhibitor', 'induction'; 5) experimental observation results including 'induced apoptosis', 'steroidogenesis', 'angiogenesis', 'migration', 'progression', 'metastasis', 'survival', 'hepatocellular carcinoma', 'lung cancer', 'carcinoma', 'therapy', 'colorectal cancer', 'cell proliferation', 'invasion', 'oxidative stress'. As shown in **Figure 5**, 9 keywords including 'migration', 'metastasis', 'hepatocellular carcinoma', 'lung cancer', 'carcinoma', 'therapy', 'colorectal cancer', 'invasion' and 'oxidative stress' burst after 2014 and continued to 2019. Among them, 'migration' (10.935), 'metastasis' (16.414), 'lung cancer' (10.691), 'therapy' (16.499), 'colorectal cancer' (10.779) and 'invasion' (12.0687) had relatively stronger citation bursts. The early studies focused on molecules that may be affected by cAMP signaling pathways, such as TNF alpha, tumor necrosis factor and necrosis factor alpha, whereas recent studies are more interested in experimental phenomena such as migration, metastasis, therapy, invasion, lung cancer and so on.

#### Analysis of co-cited references

**Figure 6:**
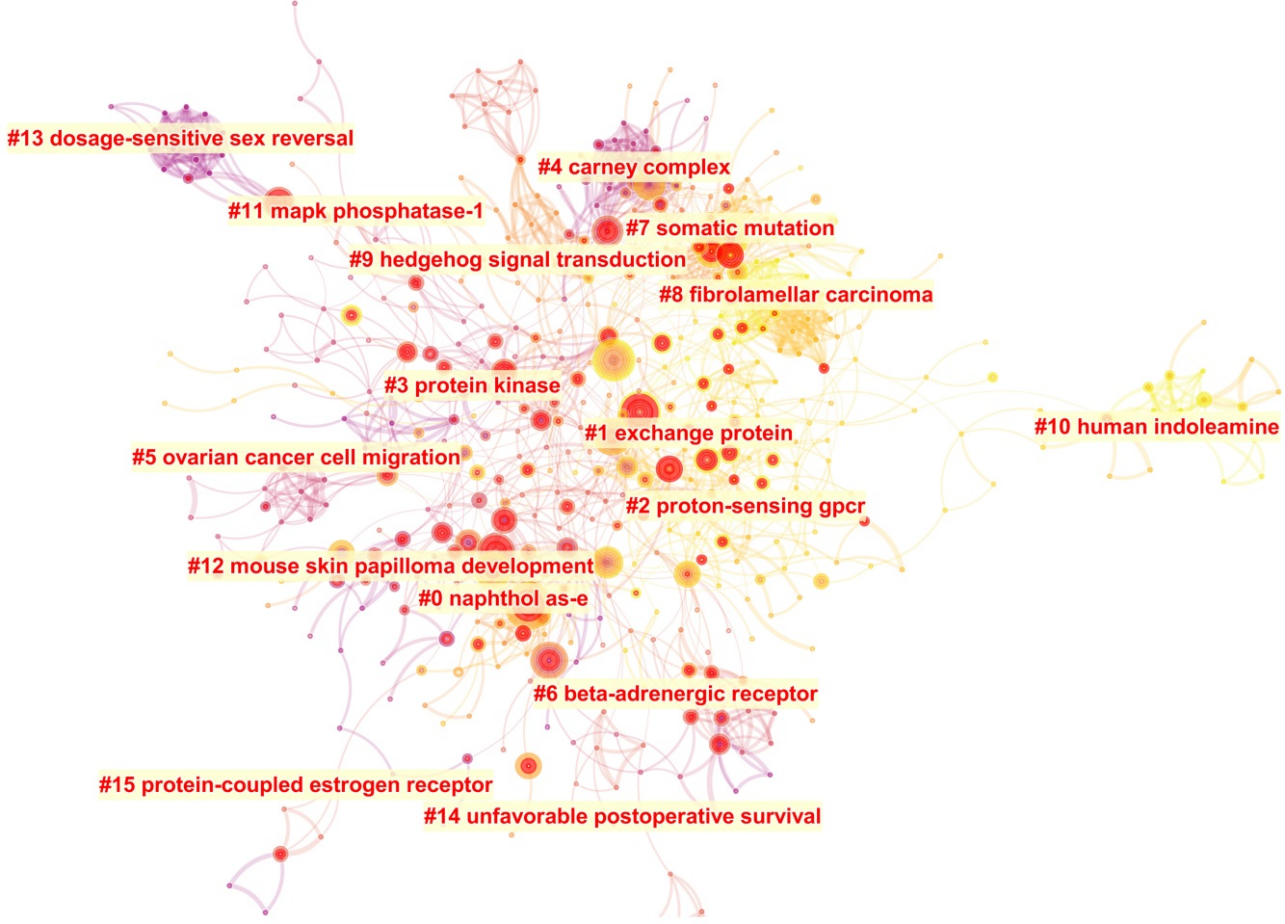
The co-cited references citation network and the 16 corresponding clusters.

The literature cited together in a group of publications is called co-cited reference, which is one of the most important indicators of bibliometrics. Based on the spectral clustering algorithm, CiteSpace can be used to construct the co-cited references' citation network map. There were 630 nodes and 1781 links on this citation network map as shown in **Figure 6.** 128 clusters were calculated, and 16 clusters listed in **Table 6** were dominant and contained a large part of these co-cited references. Modularity Q (Q value) and silhouette (S value) are two indicators to evaluate the cluster quality according to the network structure and clustering clarity. Q value is the index to evaluate the quality of the network divided into different independent modules. Generally, Q value is between zero and one. If Q value is greater than 0.300, the community structure is significant. S value is the index to evaluate the homogeneity of distinct members in the same module 28. When S value equals to 0.700, clustering is efficient and convincing. If it is above 0.500, clustering is generally considered reasonable. When S value is infinite, in usual, the number of clustering is one, the selected network may be too small to represent only one research topic. In this cluster analysis (**Table 6**), Q value is 0.777 and each S value is greater than 0.500, which means the cluster result is reliable. Each cluster label was selected by index terms from the references based on log-likelihood ratio algorithm. Cluster size represented the number of references contained in each cluster. The top 10 most citation articles and their main findings were listed in Table S1.

Burst nodes usually represent a shift in research. Top ten from the 125 references with the strongest citation bursts were listed in Table S2. Among these 125 articles, Beuschlein F et al. 39, Zhang XM et al. 40 and Horvath A et al. 41 were also listed in the top 10 references with most citations. The timeline view (Figure S4) can reflect the historical evolution trend of different clusters' cited literatures, and thus reflect the change trend of research focus. Literatures related to exchange protein (#1), proton-sensing GPCR (#2) and somatic mutation (#7) were still research hotspots until 2019. The researches related to fibrolamellar carcinoma (#8) and human indoleamine (#10) were also the focus nowadays, while the researches on naphthol AS-E (#0), carney complex (#4), ovarian cancer cell migration (#5) and beta-adrenergic receptor (#6) only attracted more attention between 2004 and 2011.

#### Research frontiers and hotspots

According to the above results, several frontiers and hotspots of research focus on cAMP signaling system in cancer field were extracted as follows:

##### cAMP signaling pathway is related to cancer cell apoptosis

Generally, cAMP signaling pathway was confirmed to be inhibited in many reported cancer tissues 17. A lot of studies have shown that if the cAMP level in cancer tissue is enhanced, the apoptosis of tumor cells can be promoted, thus inhibiting tumor growth 42,43,44. Abudoureyimu A et al. found that As_2_O_3_ induced gastric cancer BGC-823 cell apoptosis by up regulating cAMP level and down regulating PKC level 42. Daniel PM et al. proposed that the pharmacological activation of cAMP in glioblastoma (GBM) cells led to the increase of proapoptotic BIM (Bcl-2 interacting mediator of cell death) expression and apoptosis in specific types of GBM cells 17. Cheng YM et al. and Follin-Arbelet V et al. showed that cAMP could induce apoptosis in multiple myeloma cells *in vitro* 43,44. However, Hara M et al. showed that vasoactive intestinal peptide could prevent the progression of hepatocellular carcinoma (HCC) by increasing apoptosis through inhibiting cAMP / Bcl-xL pathway 45. Moreover, Safa M et al. proposed that the increase of cAMP could inhibit the As_2_O_3_-induced apoptosis in acute promyelocytic leukemia cells 18. All in all, the effect of cAMP signaling pathway on apoptosis is quite different because of different cell types. Even so, cAMP is still a possible target to inhibit the growth of many tumors.

##### cAMP signaling pathway is related to cancer cell metastasis

EPAC is one of the major downstream effectors that mediates cAMP signaling 46. EPAC consists of two subtypes, EPAC1 and EPAC2. Many studies have shown that EPAC1 is related to the metastasis of cancer cells. EPAC1 could promote the metastasis of pancreatic ductal adenocarcinoma (PDA) cells by facilitating the activation and transport of integrin βr 47,48. EPAC1 regulated the metastasis of melanoma cells through syndecan-2 translocation, heparin sulfate (HS) modification and activation of endoplasmic reticulum Ca^2+^ release via the phospholipase C (PLC)/ inositol triphosphate (IP3) receptor pathway 49,50,51. In triple-negative breast cancer (TNBC) cells, the down-regulation of EPAC1 could reduce the metastasis of TNBC cell lines 52.

However, other studies had shown that overexpression of EPAC1 in bladder cancer cell lines could significantly inhibit cell metastasis without affecting cell viability 53. The activation of cAMP/ PKA and cAMP/ EPAC1/ RAP1-GTPase pathways could inhibit the metastasis of cervical cancer cells 54. These findings suggested that cAMP signaling pathway had both positive and negative effects on cancer metastasis, which may be related to different cell types of cancer tissues. EPAC1 may be a therapeutic target to inhibit the metastasis of cancer cells.

##### cAMP signaling pathway is related to Carney complex

Carney complex (CNC) is a relatively rare autosomal dominant neoplasia syndrome. It was first reported as “the complex of myxomas, spotty pigmentation (lentigines) and endocrine overeactivity” in 1985 55. This disease is mainly caused by mutations in PRKAR1A (the gene encoding type 1A regulatory subunit of PKA) and interference with the cAMP/PKA signaling pathway. CNC patients predisposed to a variety of tumors. Endocrine tumors are the main cancers associated with CNC, including primary pigmented nodular adrenocortical disease (PPNAD), growth-hormone secreting pituitary adenoma, thyroid and gonadal tumors. Other tumors related to CNC mainly include myxomas of the heart, psamommatous melanotic schwannomas and so on 56.

Mutations in PDE11A and PDE8B were found in CNC patients with PPNAD 57,58. It was shown that the imbalance of cAMP/ PKA signaling pathway could co-regulate other signaling pathways, which participated in the occurrence of adrenocortical tumors 59. In addition, the activation of cAMP/PKA signaling pathway may play an important role in hepato-pancreato-biliary tumorigenesis of CNC patients 60. These results indicated that cAMP/PKA pathway might be the target for treatment of these lesions 61. Some researchers had proven that cAMP analogue 8-Cl-adenosine (8-Cl-ADO) could treat CNC-related tumors by inducing apoptosis through intracellular transport and metabolism inhibition 62. In summary, these studies mainly focus on the mechanism of tumor occurrence and treatment target of CNC patients, and provide ideas for the treatment of CNC-related tumors, in which cAMP signal pathway may be a breakthrough.

##### Intervening of the cAMP metabolic pathway is a potential way to treat disease or cancer

Given that the occurrence of many diseases and cancers is related to cAMP pathway, the interference of its possible target may be a promising therapeutic strategy for these diseases or cancers. For example, cAMP agonists can inhibit the growth of GBM cells 17. The combination of tricyclic antidepressants and the inhibitor of purinergic receptor P2Y12 can delay the occurrence of GBM by increasing cAMP level 63. The inhibitor of EPAC1 can slow down the remodeling of the heart and may be used as a treatment for cardiovascular diseases such as arrhythmia and heart failure 64. In addition, a study showed that EPAC1 could promote the metastasis of PDA cells and suggested that EPAC inhibitors might be a potential target of anti-metastasis drugs in PDA 41. More studies have shown that PDE inhibitors are potential drugs for the treatment of many cancers and other diseases 65-69.

**Figure 7:**
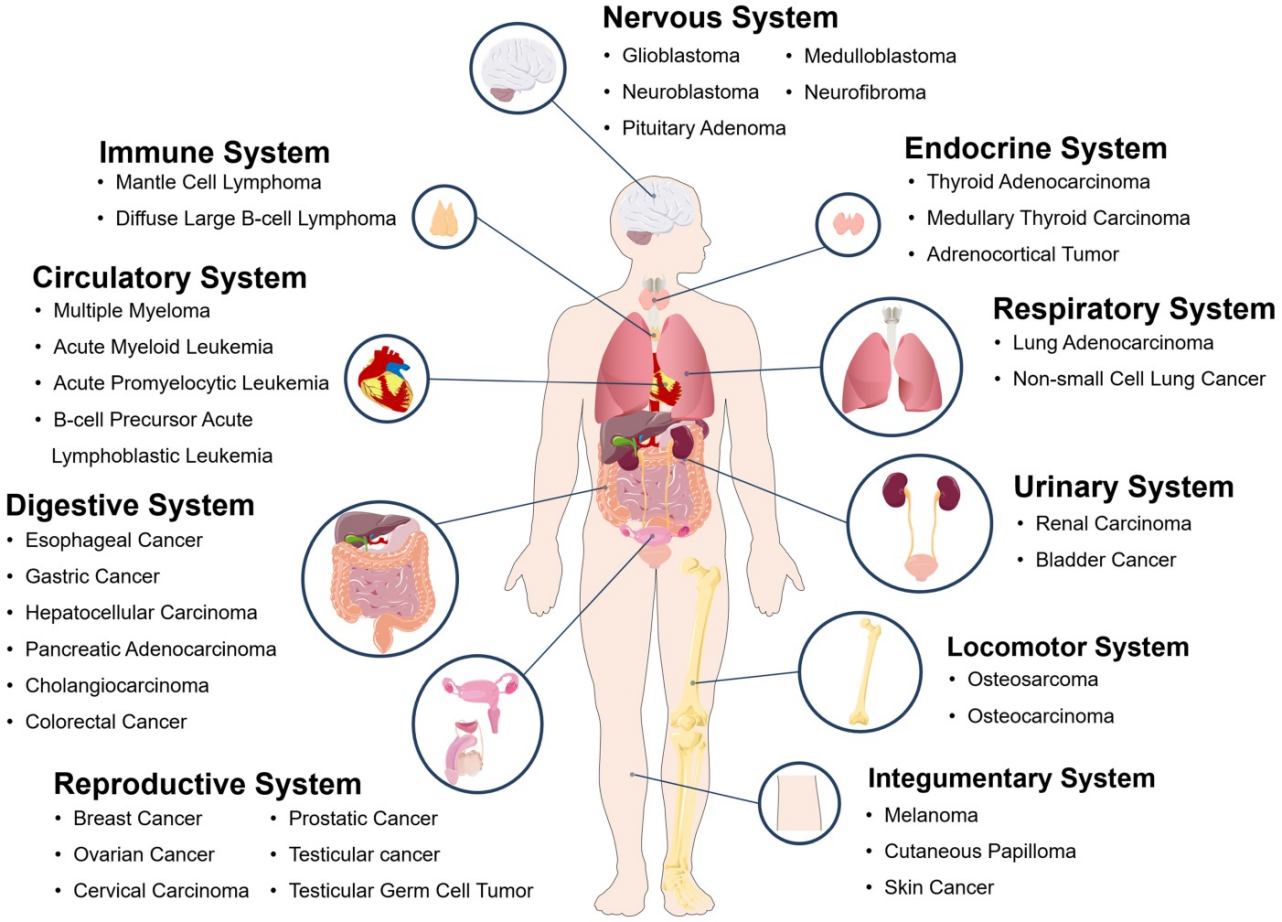
Some tumors and cancers related to cAMP signaling pathway system in various human body systems.

PDE4 inhibitors have been proved to have many functions as follows: 1) reverse the memory impairment caused by β-amyloid peptide through weakening the inflammation and apoptosis of neurons; improve memory loss and cognitive function in patients with Alzheimer's disease 70,71; 2) inhibit rheumatoid arthritis by reducing the tumor necrosis factor α (TNF-α) produced by human synovial cells 72; 3) treat airway inflammation, such as asthma and chronic obstructive pulmonary diseases through inhibiting the release of TNF-α by human peripheral blood mononuclear cells, the proliferation of lymphocytes and the production of cytokines 73; 4) treat metabolic diseases related to aging 74,75; 5) treat alcoholic fatty liver disease and nonalcoholic fatty liver disease 75-77 and so on. In addition, zardaverine, a double selective PDE3/4 inhibitor, can induce cell cycle arrest in G0/G1 phase of HCC cells by regulating cyclin 78. In addition, some studies demonstrate that CREB is an important regulatory factor in tumorigenesis and development which is suggested to be a novel target for cancer therapy 79. As an inhibitor of CREB-mediated gene transcription 80, naphthol AS-E could be used to inhibit breast cancer bone metastasis, acute leukemia cell viability, and so on 81-84. According to the publications analyzed in this study, tumors and cancers associated with cAMP signaling system in various human body systems are summarized and drawn in **Figure 7.** Therefore, the treatment targeting cAMP signaling system may be a potential universal approach for fighting against various cancers and other diseases.

### Strengths and limitations

As far as we know, this study is the first bibliometric analysis of the researches related to cAMP signaling system in cancer field. Compared with the traditional literature review, the bibliometric analysis by CiteSpace is more comprehensive and intuitive. However, this study still has some limitations. 1) This study only extracts the literature from the Web of Science Core Collection database. 2) We only selected articles published in English. 3) CiteSapce for bibliometric analysis is only based on the main information on the literature, not the full text. 4) The clustering is although reasonable in terms of Q value and S value, there are still some biases.

## Conclusion

In summary, this study helps us to understand the basic current research situation of cAMP signaling system in cancer field from 2009 to 2019. The number of related literatures published each year is relatively stable. In the past decade, USA contributed the most to the research on cAMP signaling system in cancer field, followed by China and Japan. The top three most productive journals were *J Biol Chem*, *P Natl Acad Sci* and *Nature*. Cooperation between the institutions from different countries needs to be strengthened. Lots of corresponding researches in this field are focused on the apoptosis, metastasis of cancer cell and Carney complex, suggesting that cAMP may become an ideal target for contemporary therapies for cancer and tumor diseases.

## Supplementary Material

Supplementary figures and tables.

## Figures and Tables

**Table 1:** Top ten categories of cAMP signaling system with its linkage to cancer from 2009 to 2019

Rank	Category	Count	Category	Centrality
1	Biochemistry & Molecular Biology	997	Pharmacology & Pharmacy	0.27
2	Oncology	826	Biochemistry & Molecular Biology	0.25
3	Cell Biology	823	Immunology	0.23
4	Pharmacology & Pharmacy	481	Biotechnology & Applied Microbiology	0.16
5	Endocrinology & Metabolism	478	Chemistry	0.15
6	Science & Technology - Other Topics	378	Neurosciences & Neurology	0.14
7	Multidisciplinary Sciences	359	Physiology	0.14
8	Medicine, Research & Experimental	264	Toxicology	0.14
9	Neurosciences & Neurology	254	Cell Biology	0.11
10	Chemistry	214	Hematology	0.10

**Table 2:** Top ten institutions contributed to publications on cAMP signaling system with its linkage to cancer from 2009 to 2019

Rank	Count	Institution	Country
1	65	Chinese Acad Sci	China
2	60	Shanghai Jiao Tong Univ	China
3	56	Univ Texas MD Anderson Canc Ctr	USA
4	54	Seoul Natl Univ	South Korea
5	49	Univ Calif San Diego	USA
6	46	NCI	USA
7	45	Univ Calif Los Angeles	USA
8	44	China Med Univ	China
9	39	Peking Univ	China
10	39	Eunice Kennedy Shriver Natl Inst Child Hlth & Hum	USA

**Table 3:** Top ten journals contributed to publications on cAMP signaling system in cancer field from 2009 to 2019

Rank	Citation	Cited Journal	IF (2018)	Journal Citation Reports (JCR)	Country
1	3112	Journal of Biological Chemistry	4.106	Q2	USA
2	2680	Proceedings of the National Academy of Sciences of the United States of America	9.580	Q1	USA
3	2086	Nature	43.070	Q1	England
4	2047	Cancer Research	8.378	Q1	USA
5	1785	Science	41.037	Q1	USA
6	1688	Cell	36.216	Q1	USA
7	1511	Oncogene	6.634	Q1	England
8	1416	Plos One	2.776	Q3	USA
9	1314	Molecular and Cellular Biology	2.705	Q2	USA
10	1307	Biochemical and Biophysical Research Communications	3.735	Q3	USA

**Table 4:** The top ten active authors and co-cited authors contributed to publications on cAMP signaling system in cancer field from 2009 to 2019

Rank	Top 10 productive author			Top 10 co-cited author		
	Author	Country	Count	Co-cited author	Country	Citation
1	Stratakis CA	USA	46	Livak KJ	USA	166
2	Bertherat J	France	21	Mayr B	USA	149
3	Nesterova M	France	11	Kirschner LS	USA	114
4	Horvath A	France	11	Wang Y	USA	110
5	Zhang W	China	10	Shaywitz AJ	USA	99
6	Schuller HM	USA	10	Li Y	USA	94
7	Al-Wadei HA	USA	9	Jemal A	USA	91
8	Stocco DM	USA	9	Zhang Y	China	85
9	Rubin JB	USA	9	Stocco DM	USA	82
10	Kawabata T	Japan	8	Taylor SS	USA	75

**Table 5:** The top 20 keywords

Rank	Frequency	Keywords	Centrality
1	977	expression	0.12
2	761	cAMP	0.09
3	650	activation	0.07
4	514	cancer	0.07
5	482	gene expression	0.14
6	420	phosphorylation	0.03
7	400	apoptosis	0.13
8	368	cell	0.04
9	354	protein kinase a	0.12
10	334	proliferation	0.08
11	328	growth	0.03
12	320	pathway	0.04
13	299	*in vitro*	0.06
14	294	protein	0.04
15	290	breast cancer	0.05
16	274	receptor	0.02
17	252	inhibition	0.02
18	242	gene	0.07
19	233	cancer cell	0.06
20	222	CREB	0.08

**Table 6:** The basic information of ten clusters

Cluster ID	Terms	Size	S value	Mean (Year)
0	naphthol AS-E	59	0.886	2008
1	exchange protein	55	0.845	2012
2	proton-sensing GPCR	43	0.847	2012
3	protein kinase	43	0.858	2007
4	carney complex	36	0.940	2007
5	PKA enhancement	36	0.935	2007
6	inhibitory neurotransmitter	34	0.885	2008
7	somatic mutation	34	0.904	2012
8	fibrolamellar carcinoma	32	0.966	2014
9	pharmacological target	30	0.968	2010
10	selective indoleamine-2	26	0.984	2013
11	MAPK phosphatase-1	17	0.963	2007
12	mouse skin papilloma development	16	0.937	2005
13	dosage-sensitive sex reversal	16	0.991	2004
14	unfavorable postoperative survival	12	0.994	2008
15	protein-coupled estrogen receptor	8	0.982	2007

## Abbreviations

cAMP: cyclic adenosine monophosphate
ACs: adenylyl cyclases
PDEs: phosphodiesterases
PKA: cAMP-dependent protein kinase
EPACs: exchange proteins directly activated by cAMP
CNGC: cyclic nucleotide-gated ion channels
CREB: cAMP response element binding protein
GBM: glioblastoma
BIM: Bcl-2 interacting mediator of cell death
HCC: hepatocellular carcinoma
PDA: pancreatic ductal adenocarcinoma
HS: heparin sulfate
PLC: phospholipase C
IP3: inositol triphosphate
TNBC: triple-negative breast cancer
CNC: carney complex
PPNAD: primary pigmented nodular adrenocortical disease
8-Cl-ADO: 8-Cl-adenosine
TNF-α: tumor necrosis factor α
