# Supplementary material for: Visualization and bibliometric analysis of cAMP signaling system research trends and hotspots in cancer
Source: J Cancer. 2021 Jan 1;12(2):358–70. doi: 10.7150/jca.47158 (PMC7738981; doi:10.7150/jca.47158)
Supplement: Supplementary file 1 — Supplementary figures and tables. [file jcav12p0358s1.pdf]

Supplementary Material

Visualization and bibliometric analysis of cAMP signaling system research trends and hotspots in cancer

Caoli Tang[1], Duanya Liu[1], Yongsheng Fan[1], Jun Yu[1], Cong Li[1], Jianmei Su[1*] and Chunhong Wang[1*]

[1]Department of Preventive Medicine, School of Public Health, Wuhan University, Donghu Road 115, Wuhan 430071, Hubei, China

[2]Key Laboratory of Regional Development and Environmental Response, Faculty of Resources and Environmental Science, Hubei University, Friendship Avenue 368, Wuhan 430062, Hubei, China

[*] Corresponding author at: Department of Preventive Medicine, School of Public Health, Wuhan University, Donghu Road 115, Wuhan 430071, Hubei, China

E-mail address: sujianmei@whu.edu.cn (J. Su); wchunhong027@whu.edu.cn (C. Wang)

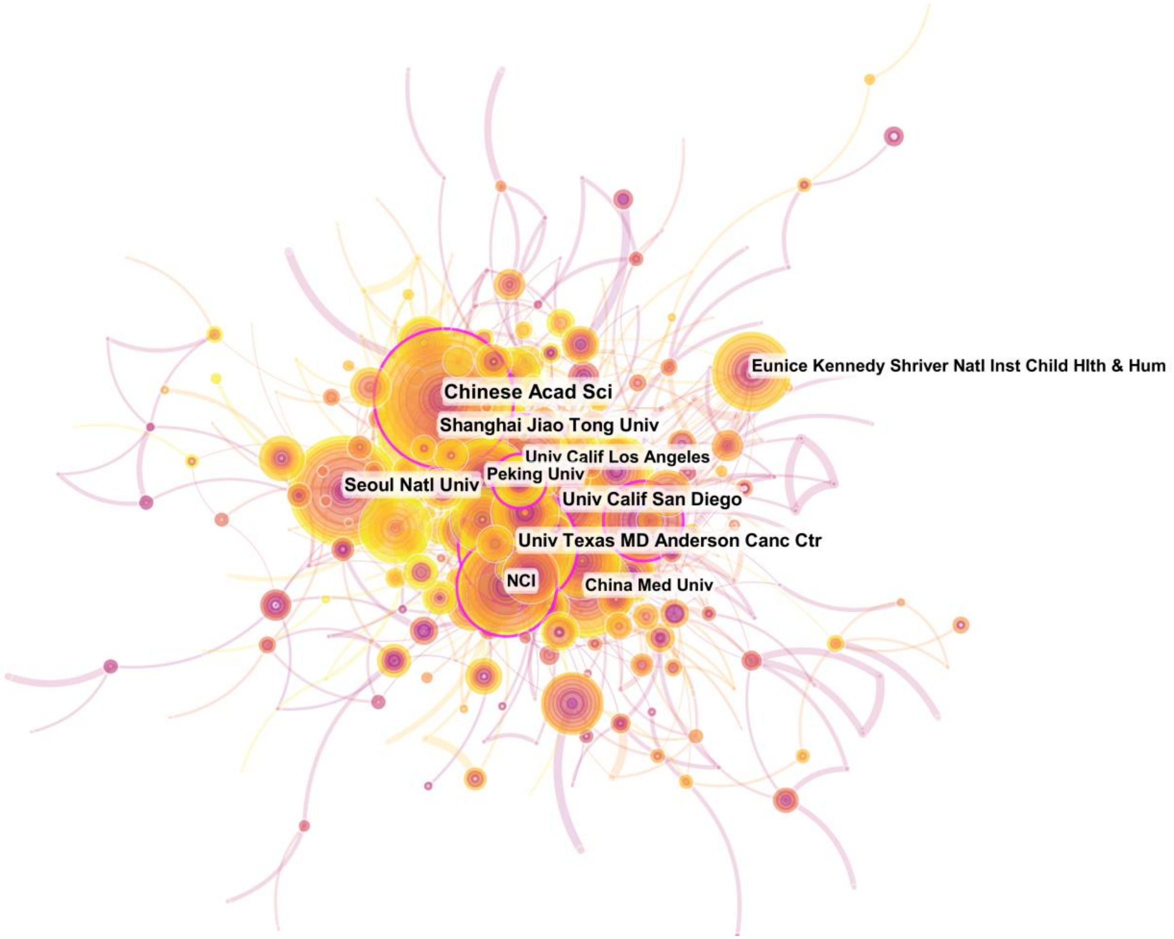

Fig S1. Collaboration network map of institutions for cAMP signaling system research in the cancer field from 2009 to 2019.

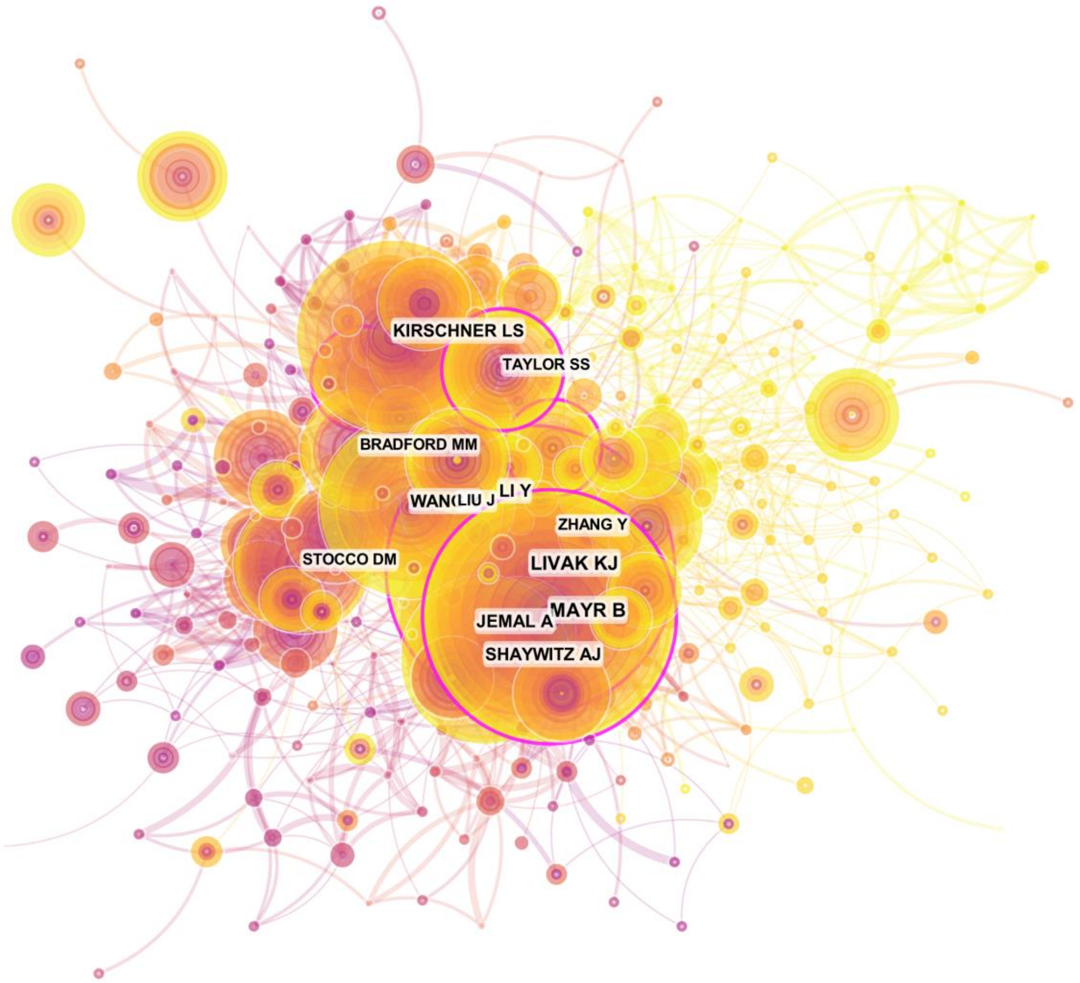

Fig S2. Author co-citation network map for cAMP signaling system research in the cancer field from 2009 to 2019.

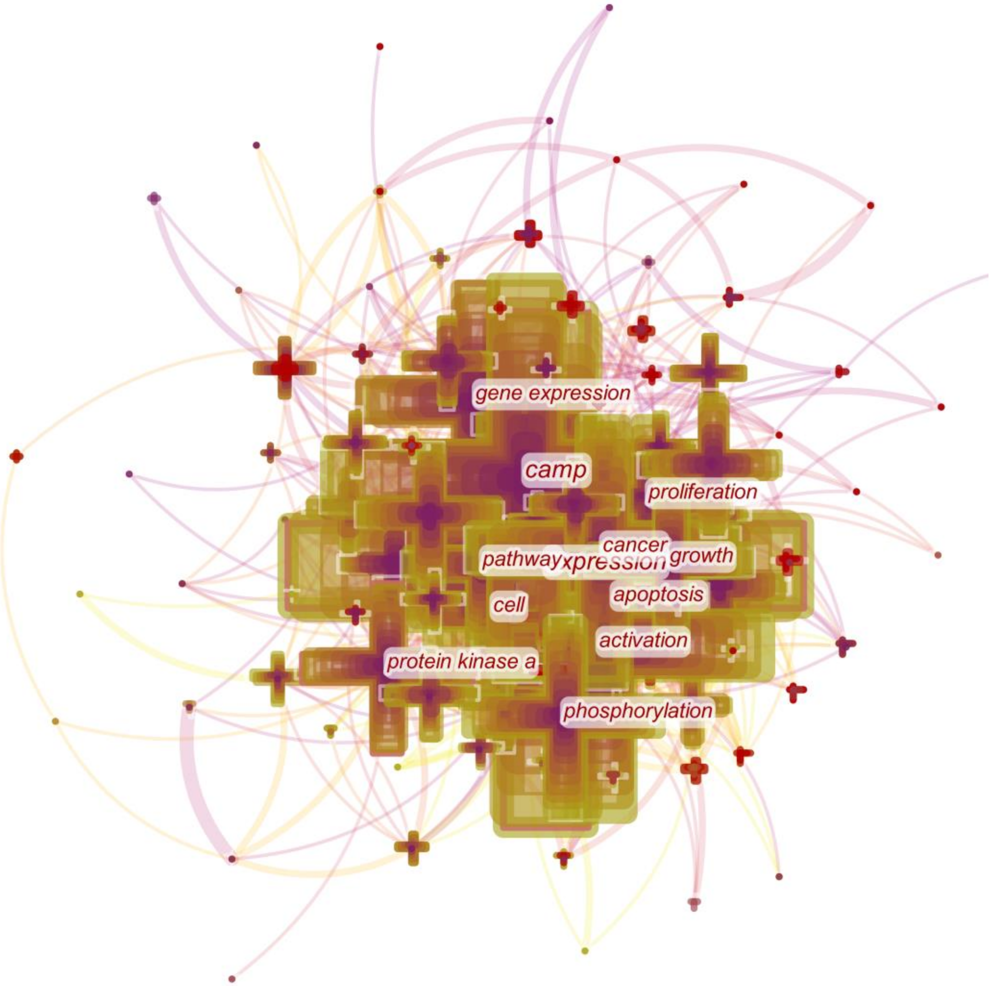

Fig S3. Visualization of keywords from the publications of cAMP signaling system in cancer field from 2009 to 2019.

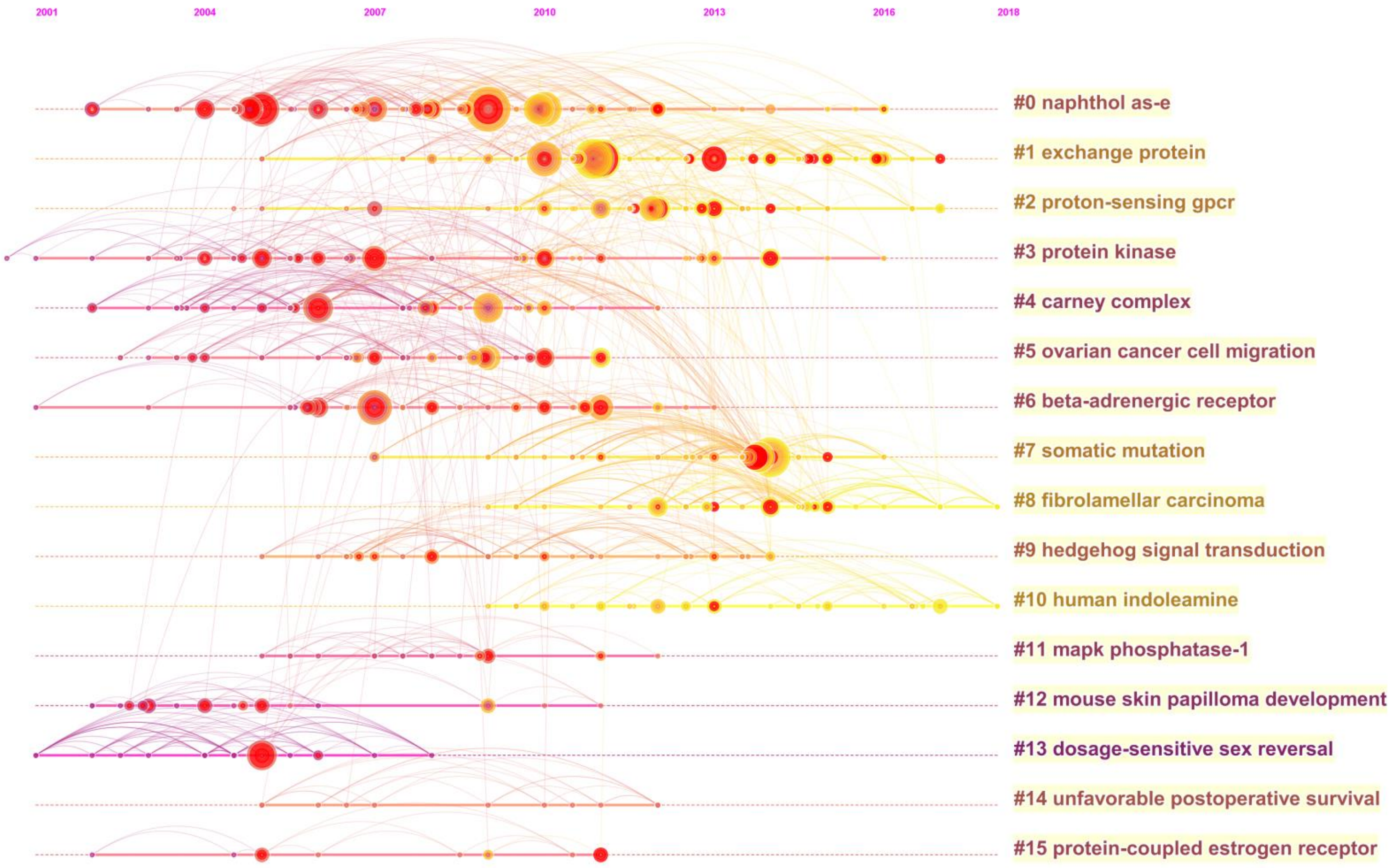

Fig S4. The timeline view of references from publications related to the research of cAMP signaling system in cancer field.

Table S1. Top 10 co-cited references about the research of cAMP signaling system in cancer field from 2009 to 2019

Rank	Citation	Co-cited Reference	Main Contents	Cluster ID
1	45	Hanahan D, 2011, CELL, V144, P646	An introduction to the original and new hallmarks of cancers, with expanding upon the functional roles and contributions made by recruited stromal cells to tumor biology.	1
2	44	Beuschlein F, 2014, NEW ENGL J MED, V370, P1019	The genetic variation of PRKACA, the main catalytic subunit of PKA, is related to the occurrence of adrenal hyperplasia and adrenal tumor.	7
3	43	Sakamoto KM, 2009, CLIN CANCER RES, V15, P2583	An introduction that CREB can play in both hematologic and nonhematologic malignancies, and it may be a target for rational therapy.	0
4	35	Zhang XM, 2005, P NATL ACAD SCI USA, V102, P4459	CREB phosphorylation alone is not a reliable predictor of target gene activation and additional CREB regulatory partners are required for recruitment of the transcriptional apparatus to the promoter.	0
5	33	Altarejos JY, 2011, NAT REV MOL CELL BIO, V12, P141	Review the research progress of CREB and cAMP-regulated transcriptional co-activators (CRTCs) in signal transduction and transcription.	1
6	33	Wen AY, 2010, I IMMUNOL, V185, P6413	An introduction to the role of the transcription factor CREB in immune function.	0
7	31	Gloerich M, 2010, ANNU REV PHARMACOL, V50, P355	Review the activation mechanism and biological functions of Epac.	1
8	29	Sugimoto Y, 2007, J BIOL CHEM, V282, P11613	An introduction to the biochemical properties of PGE receptor subtypes and physiological functions of EP subtypes.	6
9	29	Bertherat J, 2009, J CLIN ENDOCR METAB, V94, P2085	Compared with those without PRKAR1A mutation, some tumors such as myxomas, lentigines, and schwannomas are more frequently and earlier in carney complex patients with PRKAR1A mutation.	4
10	28	Horvath A, 2006, NAT GENET, V38, P794	Identify mutations in the gene encoding phosphodiesterase 11A4 (PDE11A) in individuals with adrenocortical hyperplasia that was not caused by known defects.	4

Table S2. Top 10 references from 120 bursts items

**Rank**	**Strength**	**References**	**Begin**	**End**	**Cluster**
1	9.917	Jemal A, 2011, CA-CANCER J CLIN, V61, P69	2014	2016	6
2	9.252	Beuschlein F, 2014, NEW ENGL J MED, V370, P1019	2014	2016	7
3	8.870	Zhang XM, 2005, P NATL ACAD SCI USA, V102, P4459	2009	2013	0
4	7.965	Conti M, 2007, ANNU REV BIOCHEM, V76, P481	2010	2014	3
5	7.700	Stork PJS, 2002, TRENDS CELL BIOL, V12, P258	2009	2010	40
6	7.686	Torre LA, 2015, CA-CANCER J CLIN, V65, P87	2017	2019	115
7	7.184	Conkright MD, 2003, MOL CELL, V12, P413	2009	2010	0
8	7.184	Enserink JM, 2002, NAT CELL BIOL, V4, P901	2009	2010	12
9	6.970	Thaker PH, 2006, NAT MED, V12, P939	2011	2013	6
10	6.922	Horvath A, 2006, NAT GENET, V38, P794	2009	2012	4
